# Supplementary material for: Enhanced binding of guanylated poly(A) RNA by the LaM domain of LARP1
Source: RNA Biol. 2024 Jul 17;21(1):7–16. doi: 10.1080/15476286.2024.2379121 (PMC11259064; doi:10.1080/15476286.2024.2379121)
Supplement: Supplementary_material.pdf [file KRNB_A_2379121_SM2530.pdf]

Supplementary material for

Enhanced binding of guanylated poly(A) RNA by the LaM domain of LARP1

Guennadi Kozlov[1,2], Jianning Jiang[1,2], Tyler Rutherford[3], Anne M. Noronha[3], Christopher J. Wilds[3], Kalle Gehring[1,2,*]

[1]Department of Biochemistry, McGill University, Montréal, Canada

[2]*Centre de recherche en biologie structurale*, McGill University, Montréal, Canada

[3]Department of Chemistry and Biochemistry, Concordia University, Montréal, Canada

Table of Contents

Table S1. ITC Data Collection and Analysis for LARP1 323-410 page 2

Table S2. X-ray Data Collection and Refinement Statistics page 3

Table S3. ITC Data Collection and Analysis for LARP1 323-410 Q333A page 5

Figure S1. ^{15}N-^{1}H correlation spectra of ^{15}N-labeled LARP1 (323-410) page 6

Figure S2. ITC thermograms of binding between LARP1 LaM and RNAs page 7

Figure S3. Representative electron density page 8

Figure S4. Crystal structure of LARP1 LaM with A_3GA_2 RNA oligonucleotide bound page 9

Table S1. ITC Data Collection and Analysis for LARP1 323-410

RNA	K_d, nM	Affinity relative to A_6	ΔH, kcal mol^{-1}	ΔS, cal mol^{-1} K^{-1}	Conc., µM
**AAAAAA**	250 ± 15	1	-19.9	-37.6	LaM 30 (cell), RNA 300 (syringe)
**AAAAAG**	130 ± 20	0.52	-27.5	-62.3	LaM 20 (cell), RNA 300 (syringe)
**AAAAGA**	80 ± 10	0.32	-29.5	-68.0	LaM 20 (cell), RNA 300 (syringe)
**AAAGAA**	140 ± 20	0.56	-27.7	-63.2	LaM 20 (cell), RNA 300 (syringe)
**AAGAAA**	140 ± 10	0.56	-29.3	-68.8	LaM 30 (cell), RNA 300 (syringe)
**AAAAGG**	140 ± 10	0.56	-27.4	-62.3	LaM 20 (cell), RNA 300 (syringe)
**AAAGAG**	120 ± 10	0.48	-31.2	-74.7	LaM 20 (cell), RNA 300 (syringe)
**AAGAGA**	70 ± 10	0.28	-38.3	-97.9	LaM 20 (cell), RNA 300 (syringe)
**(R_P)-AAAAA$_{PS}$A**	290 ± 30	1.16	-19.4	-36.2	LaM 25 (cell), RNA 300 (syringe)
**(S_P)-AAAAA$_{PS}$A**	17000 ± 2700	68	-23.1	-57.1	LaM 25 (cell), RNA 300 (syringe)
**UUUUUU**	1800 ± 100	7.2	-21.4	-46.6	LaM 30 (cell), RNA 300 (syringe)
**GG**	not fitted	n/a	n/a	n/a	LaM 20 (cell), RNA 290 (syringe)
**GG**	2800 ± 300	11.2	-34.1	-91.1	RNA 15 (cell), LaM 300 (syringe)
**AG**	3100 ± 200	12.4	-23.9	-56.2	LaM 30 (cell), RNA 300 (syringe)
**cyclic-di-GMP (site 1)**<br>**(site 2)**	300 ± 300<br>>10000	1.2<br>220	0<br>2.0	29.8<br>26.5	LaM 40 (cell), ligand 730 (syringe)
**3',3'-cGAMP (site 1)**<br>**(site 2)**	700 ± 300<br>>10000	2.8<br>440	0<br>4.8	28.3<br>34.4	LaM 40 (cell), ligand 750 (syringe)
**2',3'-cGAMP**	not fitted	n/a	n/a	n/a	LaM 40 (cell), ligand 860 (syringe)

Table S2. Data Collection and Refinement Statistics

**Data collection**	LaM-A_5G	LaM-A_4GA	LaM-A_3GA_2
PDB code	8EY6	8EY8	8EY7
Space group	$P2_12_12_1$	$P2_12_12_1$	$P2_12_12_1$
Cell dimensions			
a, *b*, *c* (Å)	36.52, 46.19, 58.90	36.68, 45.88, 58.86	36.91, 45.34, 59.41
Resolution (Å)	50-1.63 (1.66-1.63)[1]	50-1.30 (1.32-1.30)	50-1.35 (1.37-1.35)
R_{sym}	0.091 (0.537)	0.106 (0.427)	0.109 (0.498)
$I / \sigma I$	16.6 (1.5)	22.1 (2.2)	17.7 (1.7)
Completeness (%)	97.2 (96.3)	95.5 (65.5)	93.3 (57.6)
Redundancy	6.0 (4.8)	6.4 (2.8)	7.1 (5.0)
CC1/2[2]	0.782	0.932	0.967
**Refinement**			
Resolution (Å)	36.3 - 1.63	36.2 - 1.30	28.6 - 1.35
No. reflections	12601	23885	20855
R_{work} / R_{free}	0.208/0.242	0.183/0.192	0.186/0.225
No. atoms			
Protein	723	746	784
RNA	77	77	130
Water	33	62	81
B-factors			
Protein	30.2	22.0	16.9
RNA	51.6	35.4	21.7
Water	35.8	29.9	24.4
R.m.s deviations			
Bond lengths (Å)	0.007	0.014	0.013
Bond angles (°)	0.98	1.59	1.46
Ramachandran statistics (%)			
Most favored regions	97.7	97.7	97.8
Additional allowed regions	2.3	2.3	2.2
Disallowed regions	0.0	0.0	0.0

[1]Highest resolution shell is shown in parentheses.
[2]CC1/2 in highest resolution shell.

Table S2. Data Collection and Refinement Statistics (continued)

**Data collection**	LaM-A_5(R_P)A[3]	LaM-A_5(S_P)A[3]	LaM-U_6
PDB code	8G91	8G90	7SOW
Space group	$P4_32_12$	$P2_12_12_1$	$P2_12_12_1$
Cell dimensions			
a, *b*, *c* (Å)	53.78, 53.78, 90.45	36.95, 46.65, 59.28	36.80, 46.89, 57.90
Resolution (Å)	50-1.20 (1.22-1.20)	50-1.20 (1.22-1.20)	50-1.30 (1.32-1.30)
R_{sym}	0.104 (1.37)	0.081 (0.445)	0.060 (0.431)
$I / \sigma I$	42.4 (1.2)	35.6 (2.8)	24.7 (1.6)
Completeness (%)	99.4 (96.6)	95.2 (67.7)	99.1 (86.7)
Redundancy	22.1 (10.3)	8.6 (2.9)	7.3 (3.7)
CC1/2	0.592	0.825	0.866
**Refinement**			
Resolution (Å)	35.1 - 1.20	26.0 - 1.20	36.4 - 1.30
No. reflections	41809	31018	24922
R_{work} / R_{free}	0.178/0.189	0.192/0.209	0.182/0.192
No. atoms			
Protein	749	737	797
RNA	75	75	69
Water	106	115	66
B-factors			
Protein	17.6	17.0	20.8
RNA	33.3	29.8	29.8
Water	26.4	29.2	27.6
R.m.s deviations			
Bond lengths (Å)	0.006	0.004	0.014
Bond angles (°)	1.17	0.94	1.49
Ramachandran statistics (%)			
Most favored regions	97.7	97.7	96.8
Additional allowed regions	2.3	2.3	3.2
Disallowed regions	0.0	0.0	0.0

[3]Phosphorothioate RNA

Table S3. ITC Data Collection and Analysis for LARP1 323-410 Q333A

RNA	K_d, nM	Affinity relative to wild-type LARP1	ΔH, kcal mol^{-1}	ΔS, cal mol^{-1} K^{-1}	Conc., µM
**AAAAAG**	19000 ± 2000	146	-27.8	-73.2	LaM 30 (cell), RNA 300 (syringe)[1]
**AAAAGA**	10000 ± 6000	125	-10.2	-11.9	LaM 30 (cell), RNA 300 (syringe)[1]
**UUUUUU**	100000 ± 30000	56	-11.0	-19.2	LaM 30 (cell), RNA 600 (syringe)

[1]Data acquired on iTC200 (Malvern Instruments Ltd).

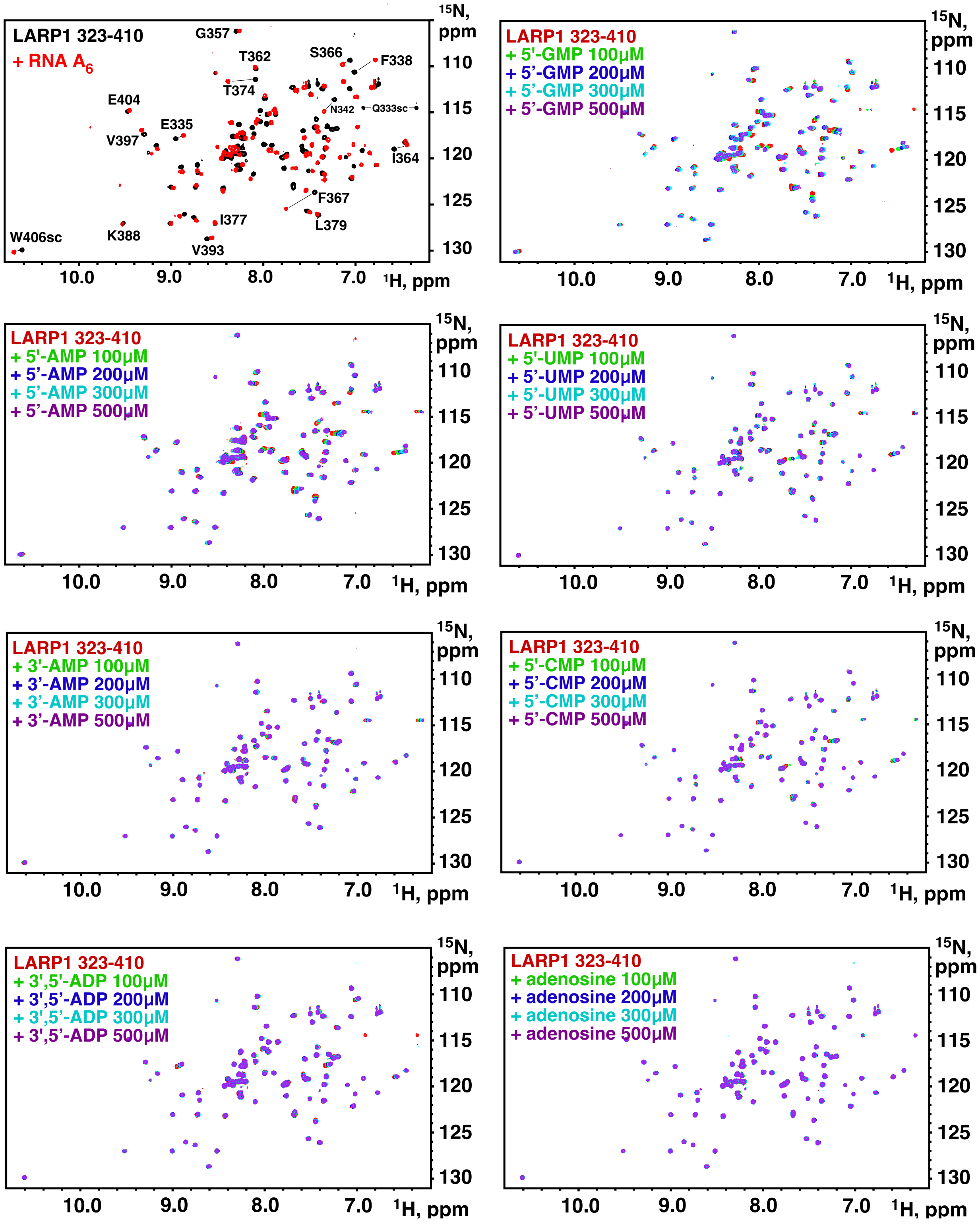

Supplementary Figure S1. ^{15}N-^{1}H correlation spectra of ^{15}N-labeled LARP1 (323-410) in the presence of RNA and single nucleotides. Peak assignments shown for A_6 are from reference [1]. sc = side chain.

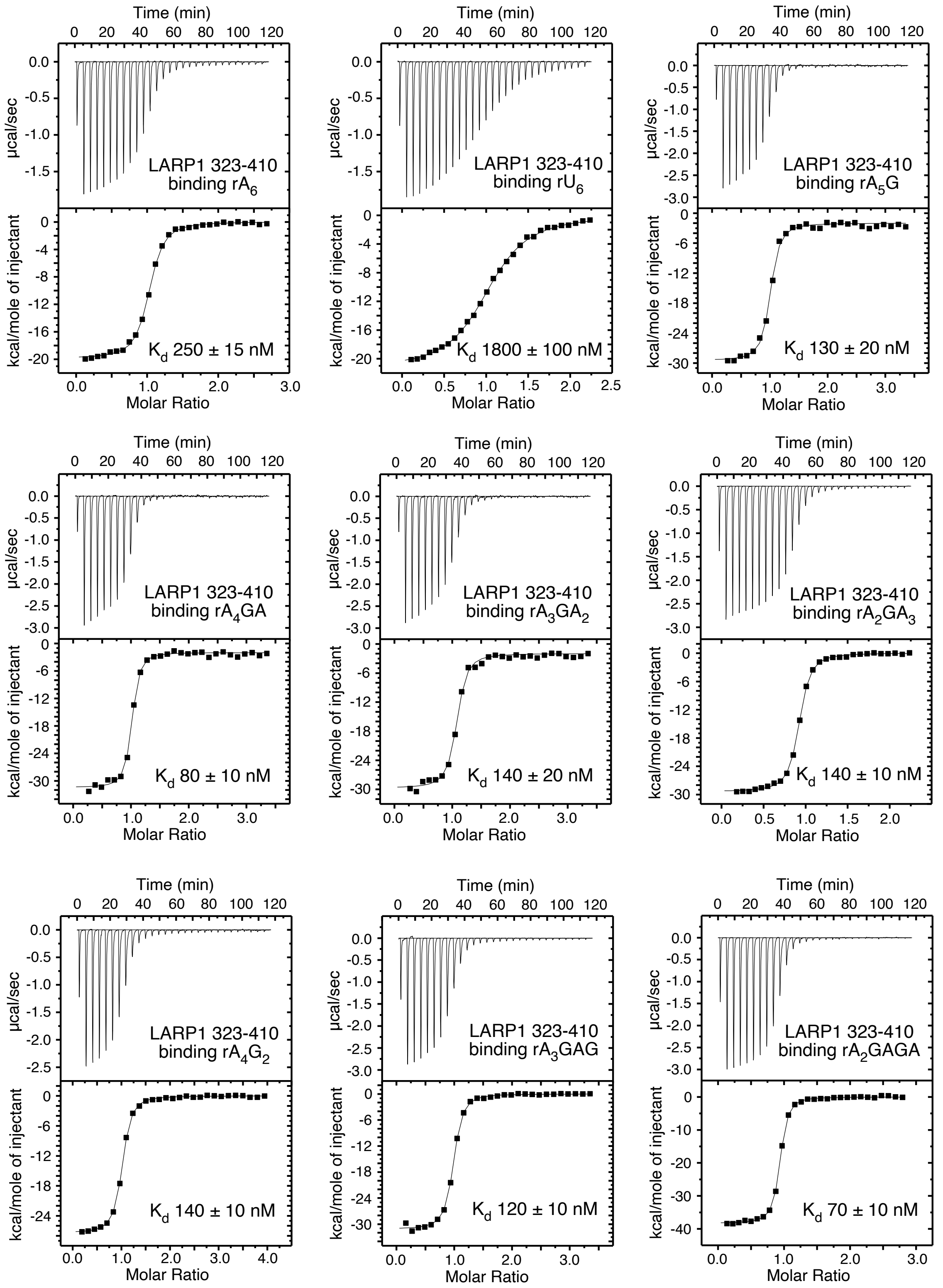

Figure S2. ITC thermograms of binding between LARP1 LaM and RNAs.

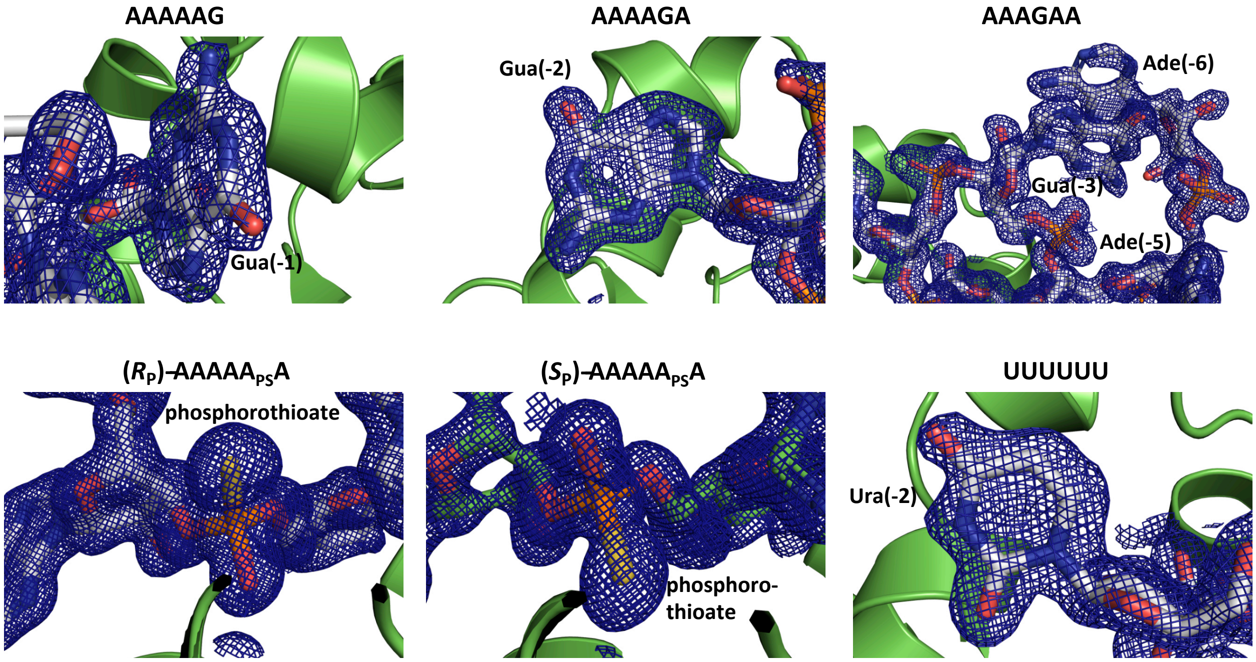

Figure S3. Representative electron density. Density is contoured at 1σ from RNA 2Fo-Fc omit maps.

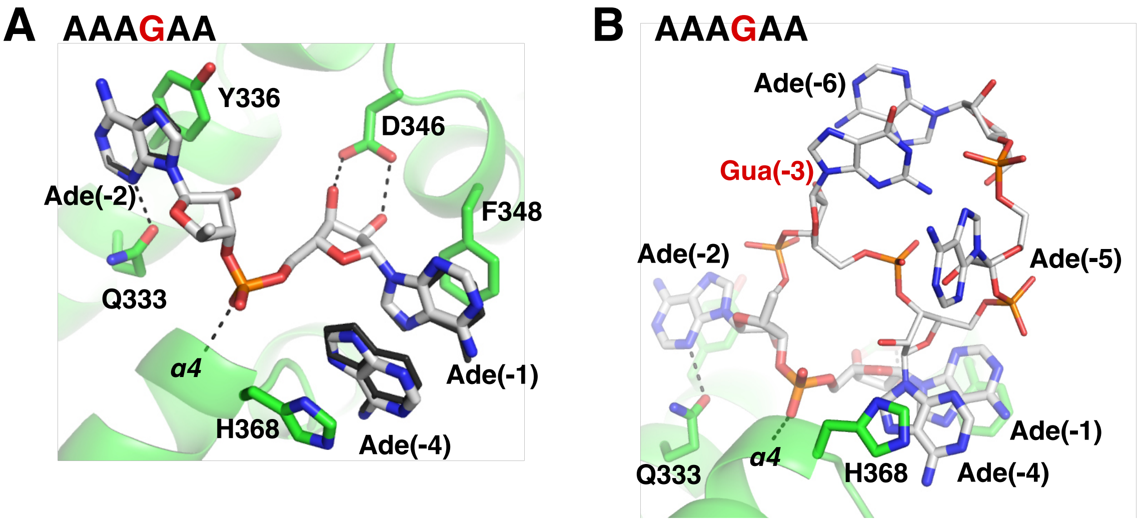

Figure S4. Crystal structure of LARP1 LaM with A_3GA_2 RNA oligonucleotide bound. **(A)** Comparison of the complex with A_6 (*black*). The two structures overlap tightly with only a small shift in the adenine ring at position (-4). (For clarity, only selected atoms are shown.) **(B)** Full model of A_3GA_2. The guanine base at position (-3) does not contact the protein but rather stacks against the base of adenylate (-6).

Literature cited

1. Kozlov, G., et al., *Structural basis of 3'-end poly(A) RNA recognition by LARP1.* Nucleic Acids Res, 2022. **50**(16): p. 9534-47.
