# Supplementary material for: Enhanced binding of guanylated poly(A) RNA by the LaM domain of LARP1
Source: RNA Biol. 2024 Jul 17;21(1):7–16. doi: 10.1080/15476286.2024.2379121 (PMC11259064; doi:10.1080/15476286.2024.2379121)
Supplement: 2nd_rebuttal.pdf [file KRNB_A_2379121_SM2534.pdf]

Reviewer 1: In their revised manuscript, Kozlov et al. made some changes in the text, but did not address any of the concerns and suggestions experimentally. While the observation that the LaM domain of LARP1 preferentially binds 3'- guanylated poly(A) RNA in vitro is potentially of interest for understanding the specificity of this important RNA-binding protein, it remains an isolated finding. Neither does the study take into account the possible contribution of other parts of the protein to RNA-binding specificity, nor does it validate the finding through independent approaches (e.g. RNA-IP, CLIP, reporter assays, comparison with datasets of guanylated mRNAs from cells).

We regret that we are not able to carry out biological studies to assess the role of tail-guanylation in LARP1 function. We have neither the facilities nor the expertise necessary to carry out tail-sequencing of mRNAs. However, that does not mean that we did not attempt to address some of the concerns experimentally. We expressed and purified two larger LARP1 constructs (residues 205-509 and 310-647). Unfortunately, they aggregated and were not suitable for ITC analysis. (The previous rebuttal document included size-exclusion chromatograms of two constructs.) We note that other groups have reported similar results. The Berman group carried out an extensive study of LARP1 (residues 310-540) and showed by circular dichroism spectroscopy that the fragment contains a large proportion of unfolded residues (Al-Ashtal et al., 2021, RNA biology 18, 248-258.)

We apologize for not more clearly pointing out the comparison of TOP and guanylated mRNAs which was previously added to the Discussion on page 11. Using lists from the Thoreen and Kim laboratories, we observed that two-thirds of TOP mRNAs could be guanylated. Conversely, 16% (62 of 390) of guanylated mRNAs were TOP mRNAs. RPSA (small ribosomal subunit protein uS2) was most frequently guanylated TOP mRNA with close to one in twenty sequences terminating with guanine or penutlimate guanine nucleotide.

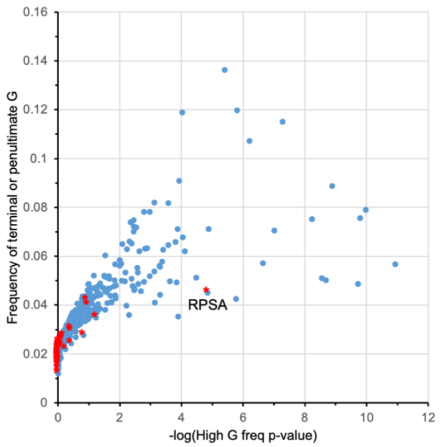

Figure 1. Scatter plot of frequency of tail-guanylated mRNAs plotted against p-value as reported by the Kim laboratory (Lim et al, 2018, Science *361*, 701-704.) TOP mRNAs identified by (Philippe et al, 2020, PNAS *117*, 5319-5328) are labeled in red.

Surprisingly, within the list of guanylated mRNAs, TOP mRNAs showed less frequent guanylation than the average (Figure 1). There are several possible explanations. It could be that guanylation of TOP mRNAs is dynamically regulated downstream of mTOR signalling. To test this, tail-sequencing would need to be repeated under different conditions of mTOR signaling. Alternatively, guanylation could be principally relevant for non-TOP mRNAs regulated by LARP1. It is also possible that extensive guanylation occurs in other classes of mRNAs through mechanisms unrelated to LARP1.

While tail-sequencing is out of the scope of the current manuscript, we were able to demonstrate additional guanine-containing ligands bind the LaM domain. The cyclic-dinucleotides, cyclic-di-GMP, 3',3'-cGAMP, and 2',3'-cGAMP, have been shown to play an important role in immune signaling and induce the synthesis of interferon. We tested whether the dinucleotides could bind to LaM in ITC experiments (Fig 3D-F). Two of the three bound with submicromolar affinity suggesting a possible link between LARP1 and the cGAS/STING pathway.
